# Clonal composition and differentiation stage of human CD30^+^ B cells in reactive lymph nodes

**DOI:** 10.3389/fimmu.2023.1208610

**Published:** 2023-07-25

**Authors:** Ralf Küppers, Bettina Budeus, Sylvia Hartmann, Martin-Leo Hansmann

**Affiliations:** ^1^ Institute of Cell Biology (Cancer Research), Medical Faculty, University of Duisburg-Essen, Essen, Germany; ^2^ Dr. Senckenberg Institute of Pathology, Goethe University Frankfurt, Medical School, Frankfurt/Main, Germany; ^3^ Frankfurt Institute of Advanced Studies, Frankfurt/Main, Germany; ^4^ Institute for Pharmacology and Toxicology, Goethe University Frankfurt, Frankfurt/Main, Germany

**Keywords:** B cells, CD30, clonal expansion, immunoglobulin genes, somatic hypermutation

## Abstract

**Introduction:**

Normal CD30^+^ B cells represent a distinct B-cell differentiation stage with features of strong activation. We lack an in depth understanding of these cells, because they are not present in peripheral blood and are typically very rare in reactive lymphoid organs. CD30^+^ B cells have been discussed as a potential precursor population for the malignant CD30^+^ Hodgkin and Reed-Sternberg cells in classical Hodgkin lymphoma. As CD30^+^ B cells can be more numerous in some cases of reactive lymphadenitis, we aimed to characterize these CD30^+^ B cells in terms of their differentiation stage and clonal composition, also as a means to clarify whether such CD30^+^ B-cell populations may represent potential precursor lesions of Hodgkin lymphoma.

**Methods:**

We microdissected single CD30^+^ B cells from tissue sections of eight reactive lymph nodes with substantial numbers of such cells and sequenced their rearranged immunoglobulin (Ig) heavy chain V region (IGHV) genes.

**Results:**

The CD30^+^ B cells were polyclonal B cells in all instances, and they not only encompass post-germinal center (GC) B cells with mutated IGHV genes, but also include a substantial fraction of pre-germinal center B cells with unmutated IGHV genes. In five of the lymph nodes, mostly small clonal expansions were detected among the CD30^+^ B cells. Most of the expanded clones carried somatically mutated IGHV genes and about half of the mutated clones showed intraclonal diversity.

**Discussion:**

We conclude that in human reactive lymph nodes with relatively many CD30^+^ B cells, these cells are a heterogenous population of polyclonal B cells encompassing activated pre-GC B cells as well as GC and post-GC B cells, with some clonal expansions. Because of their polyclonality and frequent pre-GC differentiation stage, there is no indication that such cell-rich CD30^+^ B-cell populations represent precursor lesions of Hodgkin lymphoma.

## Introduction

The tumor necrosis family receptor superfamily (TNFRSF) member TNFRSF8, which is commonly called CD30, is considered as an activation marker for B and T cells (1). CD30-positive B cells are not present in the peripheral blood of humans, and they typically account for less than one percent of B cells in reactive lymph nodes or tonsils (1, 2). CD30^+^ B cells can be found both within germinal centers (GC) as well as in extra-follicular areas of secondary lymphoid organs (1, 3), The rarity of these cells is a main reason why we know relatively little about them. In a prior study, we flow-cytometrically isolated extra-follicular and GC CD30^+^ B cells from human tonsils and performed a molecular analysis of these cells (2). The analysis revealed that practically all CD30^+^ GC B cells carried somatically mutated immunoglobulin (Ig) V genes, indicating that they are normal participants of GC reactions (2). Also, most extra-follicular tonsillar CD30^+^ B cells harbored mutated IgV genes, showing a GC derivation of these cells. However, some of these cells were unmutated, indicating a heterogeneity in the origin of extra-follicular CD30^+^ B cells (2). Global gene expression profiling of the tonsillar CD30^+^ B cells confirmed that these cells are highly active cells, with upregulation of hundreds of genes in comparison to naive and memory B cells, as well as conventional GC B cells (2). A striking hallmark of GC and extra-follicular CD30-expressing B cells was a strong MYC expression and MYC activity signature, and hints for an interaction with T helper cells. As murine studies revealed that MYC^+^ GC B cells represent centrocytes that are positively selected and preparing to return to the dark zone of the GC (4, 5), this suggests that human CD30^+^ GC B cells represent the human equivalent of these cells and hence the small subset of centrocytes selected to return to the dark zone for further rounds of proliferation and IgV gene mutation (2). Extra-follicular CD30^+^ B cells may also have obtained stimuli form antigen and T helper cells, inducing their proliferation and MYC expression in extra-GC location (2).

Normal CD30^+^ B cells are not only of interest as peculiar and distinct subsets of human B cells, but also because of a potential relationship to lymphoma cells. In classical Hodgkin lymphoma (HL), the Hodgkin- and Reed-Sternberg (HRS) tumor cells are always CD30-positive (6). Indeed, their CD30 expression is a main diagnostic marker (6), and is nowadays also of therapeutic relevance, as treatment of HL patients with a toxin-conjugated anti-CD30 antibody (Brentuximab vedotin) is a promising targeted treatment approach at relapse (7). CD30 expression is also a hallmark of the lymphoma cells in primary mediastinal B-cell lymphoma, and is also seen in about 10% of cases of diffuse large B-cell lymphomas other than the primary mediastinal subgroup (6, 8). It has been speculated that HRS cells (and perhaps also CD30^+^ lymphoma cells in B cell Non-Hodgkin lymphomas) may derive from normal CD30^+^ B cells, and our comparative gene expression profiling study mentioned above indeed revealed that normal CD30^+^ B cells are the B-cell subset most similar in its gene expression pattern to HRS cells of classical HL, when also considering naive B cells, memory B cells, conventional GC B cells and plasma cells (2).

In several types of B-cell malignancies, lymphoma precursor lesions are known which will in some individuals later develop into a frank B-cell malignancy. The prototypical examples of this are monoclonal B-cell lymphocytosis as a precursor lesion of chronic lymphocytic leukemia, and follicular lymphoma in situ, which may develop into follicular lymphoma (9, 10). These precursor lesions already show some lymphoma/leukemia-specific somatic mutations, and are monoclonal B-cell proliferations, supporting a pre-malignant identity (9, 10). As some reactive lymph nodes harbor a considerable number of CD30^+^ B cells, we wondered what the characteristics of these CD30^+^ B-cell populations is in terms of their clonal composition and differentiation stage, and whether they may sometimes represent precursor lesions of classical HL. To address this issue, we microdissected single CD30^+^ B cells from eight reactive lymph nodes with a relatively large number of CD30-positive cells and amplified and sequenced their rearranged IGHV genes, as this is an elegant approach to determine the clonal composition of these cells and their differentiation stage.

## Materials and methods

### Selection and characterization of lymph node biopsies

Formalin-fixed and paraffin-embedded (FFPE) biopsies of reactive lymph nodes for which parts were also available as frozen tissue were stained for CD30 with the anti-CD30 monoclonal antibody BerH2 (Dako, Hamburg, Germany) to identify lymph nodes with a substantial number of CD30^+^ cells. Five of the eight cases included in the IGHV gene analysis were further characterized by double stainings for CD30 (clone EP154, Diagnostic Biosystems, Pleasanton, CA, USA, at dilution 1:100) combined with staining for PAX5 (clone DAK-PAX5, Dako, at dilution 1:100), CD138 (clone MI15, Dako, at dilution 1:100), or MUM1/IRF4 (clone MUM1P, Dako, at dilution 1:100). The double stainings were performed using the Vecta Fluor Duet Double Labeling Kit DK-8828, Vector Laboratories, Newark, CA, USA.

### Laser microdissection and pressure catapulting of CD30^+^ B cells

Frozen lymph node sections positioned on membrane-covered microdissection slides were immunostained for CD30 with the anti-CD30 antibody BerH2 (Dako). Single CD30^+^ B cells were microdissected into 20 µl of 1 x PCR buffer using the PALM Robot MicroBeam laser microdissection system (Zeiss, Oberkochen, Germany) as previously described (11). Microdissected pieces of membrane not covered by tissue and tubes only carrying PCR buffer served as negative controls.

### IGHV gene amplification, sequencing, and evaluation from single cells

Microdissected cells were lysed with proteinase K (0.25 mg/mL) for 3 hours at 55°C. The enzyme was inactivated by heating at 95°C for 10 minutes. For each set of cells several tubes with catapulted membrane without tissue and empty tubes were analyzed in parallel as negative controls. Some sets in which products appeared in negative controls were discarded. Rearranged IGHV region genes were amplified from DNA of the single microdissected cells in a semi-nested PCR using subgroup specific IGHV framework region 1 (FR1) primers combined with IGHJ primer mixes according to an established protocol (11). Expand high fidelity polymerase mixture (Roche) was used in the first round of PCR and Taq DNA polymerase in the second round. PCR products were agarose-gel-purified and directly sequenced by Sanger sequencing on an ABI 3010 sequencer (Applied Biosystems, Darmstadt, Germany), using the IGHV region primers for sequencing. For each sequence, the corresponding IGHV gene was determined by rBLAST (https://github.com/mhahsler/rBLAST) using the database for all human IGHV genes from IMGT (https://www.imgt.org/). Clones were determined via R in the following procedure: All sequences with the same IGHV gene, maximal 5% length difference in complementarity determining region III (CDRIII) and > 90% nucleotide sequence identity among all CDRIII sequences are included into a clone. An IGHD gene was assigned only if at least 7 bases in a row fit to the candidate germline sequence. Sequences with only a single nucleotide difference to the germline gene were counted as unmutated, as PCR errors in the first rounds of the reaction or hypermutation-independent mutation events may potentially account for this.

## Results

### Selection and characterization of lymph nodes with numerous CD30^+^ B cells

**Figure 1:**
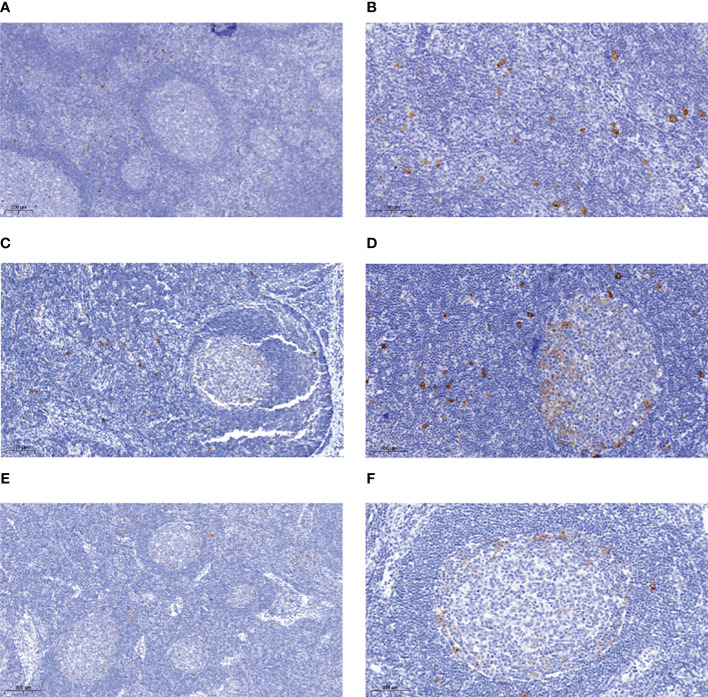
Immunohistochemical analysis of reactive lymph nodes for CD30-expressing lymphocytes. Tissue section of FFPE biopsies of reactive lymph nodes rich in CD30^+^ B cells were stained for CD30 using the BerH2 antibody. Exemplary stainings for four cases are shown. **(A)** case 2, in which most CD30^+^ B cells are in interfollicular regions. **(B)** case 2, higher magnification. **(C)** case 3 in which the CD30^+^ cells are evenly distributed between GC and interfollicular regions. **(D)** case 5 in which the CD30^+^ cells are evenly distributed between GC and interfollicular regions. Note the predominant location of CD30^+^ B cells at the outer border of the GC. **(E)** case 7 with mainly intra-GC localization of CD30^+^ cells. **(F)** case 7 higher magnification. Note the predominant location of CD30^+^ B cells at the outer border of the GC. A size bar is indicated in each picture in the lower left area.

About 50 reactive lymph nodes, for which both FFPE as well as fresh frozen parts were available, were stained for CD30, using the FFPE material for screening. Thirteen lymph nodes showed relatively many CD30^+^ cells (**Figure 1**). Eight of the lymph nodes with good quality frozen material were selected for molecular analysis (**Supplementary Table 1**). Typically, most CD30^+^ cells were seen in extra-follicular areas, but some also within GC, mainly at the outer borders of the GCs. As the distinction between these areas was sometimes not easy in the frozen tissue sections, and as we were interested in the overall CD30^+^ B-cell population, we did not separate GC from non-GC CD30^+^ B cells for the IGHV gene sequence analysis.

**Figure 2:**
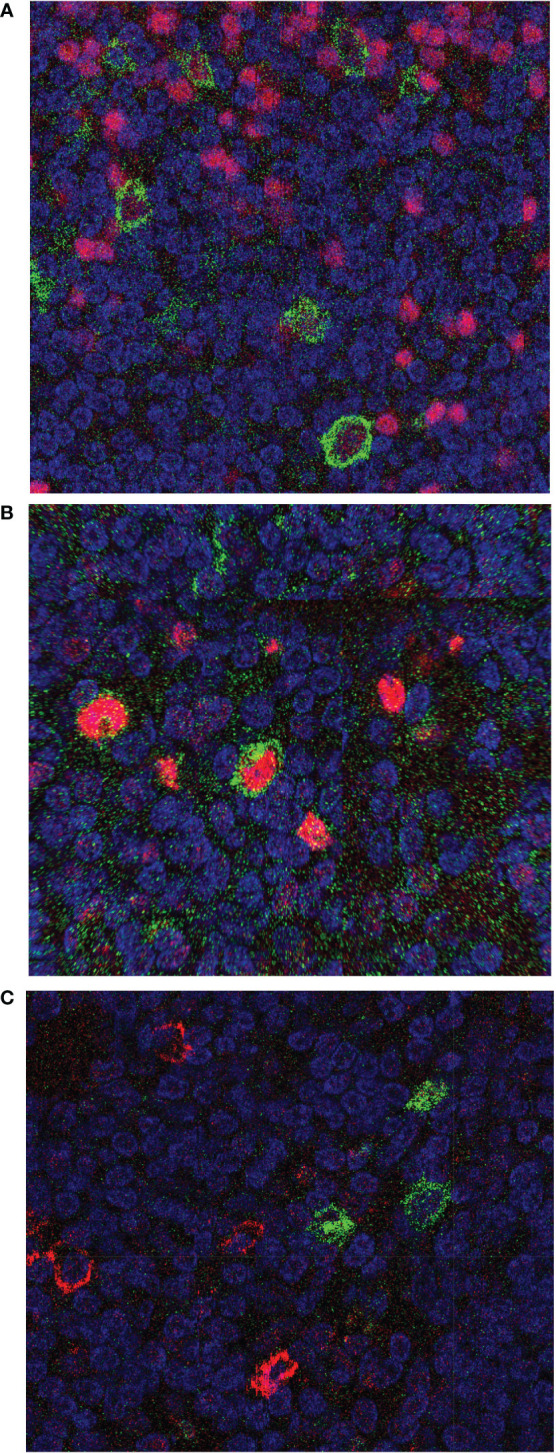
Immunohistochemical double stainings of CD30 combined with PAX5, MUM1, or CD138. Tissue section of FFPE biopsies of the reactive lymph node from case 3 were stained for CD30 using the BerH2 antibody, combined with staining for **(A)** PAX5, **(B)** MUM1, or **(C)** CD138. CD30 is always depicted in green color, the second staining in red color. In **(A)** all CD30^+^ cells express PAX5, i.e., are B cells. In **(B)** the centrally located CD30^+^ B cell is MUM1-positive, and additional MUM1-expressing cells lacking CD30 staining are visible. In **(C)** it is visible that CD138 and CD30 expression is mutually exclusive.

For five of the cases (1, 3, 4, 5, and 8) we performed selected double stainings with FFPE tissue sections to further characterize the CD30^+^ cells. The CD30^+^ cells showed a variable fraction of PAX5-positive cells (**Table 1**; **Figure 2A**). PAX5 is a general B cell marker, and the CD30^+^PAX5^-^ cells likely represent CD30^+^ T cells, but may also include some B cells with a too low PAX5 expression to be clearly detectable in the double staining. The CD30^+^ B cells consistently lacked CD138 expression, in line with prior studies reporting that CD30^+^ B cells lack key features of plasma cells (**Table 1**; **Figure 2C**) (2, 12). MUM1 expression was variable on CD30^+^ cells. In four of the five cases studied here, half to nearly all of the CD30^+^ cells were MUM1^+^ (**Table 1**; **Figure 2B**). This fits to earlier reports that CD30^+^ B cells frequently express this transcription factor (3, 13, 14). As EBV infection of B cells can induce CD30 expression in the latency III program, we also studied two of the lymph nodes by EBER *in situ* hybridization, but no EBER-expressing cells were identified in the sections analyzed (data not shown).

**Table 1:** Phenotypic characterization of CD30^+^ cells.

Case	Region scored in analysis	CD30^+^ cells positive for		
		CD138	PAX5	IRF4/MUM1
1	interfollicular	0/15	10/10	16/18
3	GC	0/3	10/10	6/7
	interfollicular	0/8	3/10	6/12
4	interfollicular	0/6	4/7	7/10
5	interfollicular	0/7	4/9	15/20
8	interfollicular	0/7	0/5	2/4

### IGHV genes of single human CD30^+^ B cells

For the study of the clonal composition and differentiation stage of normal human CD30^+^ B cells in reactive lymph nodes, we microdissected over 100 single CD30^+^ cells from immunostained frozen tissue sections from each of eight lymph nodes and amplified their rearranged IGHV genes in a two-rounded semi-nested PCR, using IGHV subgroup-specific FR1 primers and IGHJ primers. The resulting PCR products were directly sequenced. As we did not perform double staining with a B-cell marker for the cell isolation from frozen tissue sections, some CD30^+^ cells are likely T cells, as also indicated from our exemplary CD30/PAX5 double staining described above. Typically, B cells are the predominant population of CD30^+^ lymphocytes (3, 14), and microdissected CD30^+^ T cells would simply remain negative in the IGHV gene PCR.

From 20 to 36 cells per case mostly one and in a few instances two IGHV gene rearrangements were obtained (**Table 2**). The vast majority of IGHV genes were potentially functional, as expected, and only a few non-productive rearrangements were obtained (**Supplementary Tables 2**-**9**). In a few instances two potentially functional IGHV genes were amplified from one cell sample (**Supplementary Tables 2**-**9**). This could be due to inclusion of part of a second B cell during microdissection together with the CD30^+^ B cells. However, as only few cells showed this pattern, none of the features described for the CD30^+^ B cells in our analysis is compromised by this. An efficiency of about 20-25% to obtain the productive IGHV gene rearrangement of the microdissected B cells analyzed is well in line with similar prior studies (15–17) and can be explained by several reasons: a) often, part of the nucleus of a cell will be missing in the tissue section, b) not always will the cell digestion free the DNA of the single gene copy of a rearrangement from histones and other proteins, hindering its amplification by PCR, c) sometimes, somatic mutations at the primer binding sites will prevent successful amplification.

**Table 2:** IGHV gene analysis of CD30^+^ B cells.

Case	No. of CD30^+^ B cells with IGHV amplificates	Fraction of cells with mutated IGHV genes (%)	Average mutation frequency of mutated IGHV genes* (%)	CD30^+^ B-cell clones	
				unmutated	mutated
1	28	46.4	6.6	0	0
2	36	61.1	3.2	2x 2 cells	2x 2 cells, 1x 3 cells
3	28	85.7	6.9	0	1x 2 cells
4	24	54.0	9.1	0	1x 5 cells
5	23	56.0	5.8	0	0
6	20	75.0	6.7	0	2x 2 cells
7	30	70.0	5.7	0	0
8	33	69.7	7.0	1x 2 cells	1x 3 cells, 3x 2 cells

*IGHV rearrangements with only a single nucleotide difference to the IGHV germline gene (0.5% difference) were also considered as unmutated.

The IGHV gene usage of the productive IGHV region genes amplified form the CD30^+^ B cells is divers. As expected for normal B-cell populations, usage of the large IGHV3 subgroup is predominant, followed by usage of IGHV4 and IGHV1 subgroup members, and only rare usage of IGHV2 or IGHV5 subgroup members (**Supplementary Tables 2**-**9**). No single IGHV gene was prominently used. In only two lymph nodes, there seems to be over usage of a single IGHV gene among the productive rearrangements. This is case 3, in which 6 of 26 cells with productive IGHV genes used the *IGHV3-30* gene (**Supplementary Table 4**), and case 7, in which 9 of 29 B cells with productive IGHV genes used the *IGHV1-69* gene (**Supplementary Table 8**).

Between 46-86% of the cells carried somatic mutations in their rearranged IGHV genes (**Table 2**), showing that in each of the lymph nodes studied, the population of CD30^+^ B cells is heterogenous in its differentiation stage, with half to nearly 90% of the cells having a GC experience and hence represent GC or memory B cells. Still, in each case over ten percent to about half of the cells are pre-GC B cells with unmutated IGHV genes, and hence can be considered as activated naive B cells. For the somatically mutated IGHV genes, the average mutation frequencies ranged between 3.2-9.1%, with most cases having average mutation frequencies of 6-7% (**Table 2**). This is a range that is typical for class-switched memory B cells (18).

**Figure 3:**
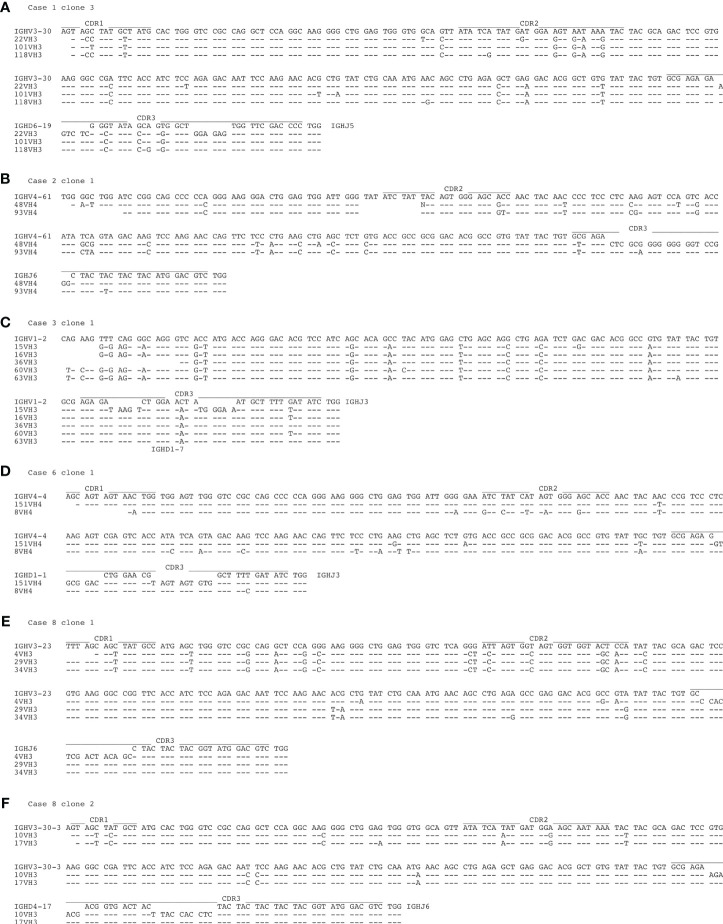
Intraclonal IGHV gene diversity in expanded CD30^+^ B-cell clones. Shown are the IGHV gene sequences of the six B-cell clones with mutated IGHV gene sequences and intraclonal diversity. **(A)** case 1, clone 3, **(B)** case 2, clone 1, **(C)** case 3, clone 1, **(D)** case 6, clone 1, **(E)** case 8, clone 1, **(F)** case 8, clone 2. The locations of the complementarity determining regions (CDR) 1-3 is indicated. Lines indicate sequence identity to the upper sequence. In two instances (case 2 clone 1, case 8 clone 1) the IGHD genes could not be identified.

In five of the eight lymph nodes investigated mostly small clones with 2-3 members were detected among the CD30^+^ B cells (**Table 2**). Overall, 14 clones were found. In case 4, a single clone with five members was identified among 24 informative B cells. Three of the clones carried unmutated IGHV genes and did not show intraclonal diversity. Eleven clones carried somatically mutated IGHV genes. Of these, five lacked intraclonal diversity, but six clones showed sequence variation among clone members (**Figure 3**). These observations indicate that not only GC-experienced CD30^+^ B-cell clones underwent (as expected) clonal expansions, but that also pre-GC CD30^+^ B cells are at least partly proliferating.

## Discussion

In reactive lymph nodes a few CD30^+^ lymphocytes can typically be identified (19). These have since long been considered as activated B or T cells (19). Sometimes the number of such cells is increased, and this may then afford a differential diagnosis to classical HL, where the B cell-derived HRS cells are also CD30^+^ and typically account for only one or a few percent of cells in the lymphoma tissue. HRS cells derive from GC-experienced B cells but have largely lost their B-cell gene expression program (20, 21). Although the normal CD30^+^ B cells have largely retained their B-cell program, CD30^+^ B cells show remarkable similarities to HRS cells (2). This has led to the speculation that HRS cells may derive from CD30-positive B cells (2). Moreover, in some lymphoid malignancies, precursor states of leukemia or lymphoma cells have been identified, that are already monoclonal and already carry some genetic alterations that are also recurrent mutations in the malignant counterparts (9, 10). On the basis of these observations, we wondered whether lymph nodes that were diagnosed as reactive conditions with substantial numbers of CD30^+^ B cells may at least sometimes present oligo- or monoclonal expansions of GC-experienced B cells. We used IGHV gene sequencing from single microdissected CD30^+^ B cells as a means to determine the clonal composition and the differentiation stage (pre-GC versus GC-experienced) of the CD30-expressing B cells.

The analysis of CD30^+^ B cells from eight reactive lymph nodes revealed that in each of the cases the CD30^+^ populations were polyclonal. In five of the samples, a few small clones of mostly 2-3 members were identified among the CD30^+^ B cells, which is in line with the finding that the highly activated CD30^+^ B cells show features of proliferating cells, including a strong MYC target gene signature (2). It is likely that the clones are in reality considerably larger, as only 20-36 CD30^+^ cells were characterized per lymph node. However, this does not affect the conclusion that the populations are polyclonal. Only in one of the cases, a somewhat larger clone with five members among a total of 24 informative B cells was obtained (case 4). This is clearly different from situations such as monoclonal B-cell lymphocytosis or follicular lymphoma in situ, where large monoclonal expansions of B cells are detected (9, 10). Thus, we conclude that lymph nodes with many CD30^+^ B cells are typically not monoclonal expansions of putative HRS precursor cells. We cannot exclude that in the development of classical HL such intermediate stages exist, but such situations are not the rule for reactive lymph nodes with large numbers of CD30^+^ B cells. Still, CD30^+^ B cells may be precursors of lymphomas with CD30^+^ lymphoma B cells. Considering their highly activated and proliferative state, they may indeed be at an increased risk for malignant transformation. The observation that CD30^+^ lymphoma B cells in HL, primary mediastinal B-cell lymphoma and a subset of further high-grade lymphomas in nearly all cases carry somatically mutated IGV genes (21–25), indicates that also for CD30^+^ B cells, a GC passage with the particularly high proliferative activity of GC B cells and the mutagenic processes of somatic hypermutation and class-switching pose a specifically high risk for malignant transformation (26), so that most CD30^+^ B-cell lymphomas derive from GC-experienced B cells.

As we still have little knowledge about the characteristics of normal CD30^+^ B cells, our analysis provides valuable additional insights into these cells. In each of the cases, the CD30^+^ B cells were a mixture of pre-GC B cells with unmutated IGHV genes and GC or post-GC B cells with mutated IGHV genes. The B cells with unmutated IGHV genes likely represent activated naive B cells, although it cannot be excluded that some of them are memory B cells generated after T-cell interaction in the primary focus reaction without entering into a GC reaction. Such pre-GC T-dependent memory B cells with unmutated IGV genes have been described in mice (27, 28). The fraction of CD30^+^ unmutated B cells varied from about 15 to over 50%. This indicates that we are here observing immune reactions in which on the one hand naive B cells have been activated, and on the other hand GCs are induced and/or memory B cells are reactivated and acquire a CD30^+^ B-cell program. The existence of CD30^+^ GC B cells is apparently a regular phenomenon, as these cells seem to represent the small and transient light zone GC B-cell subset that upregulates MYC and prepares for return to the dark zone for a further round of proliferation and mutation (2, 4, 5). However, why activated naive B cells and/or reactivated memory B cells in extra-follicular location in some instances upregulate CD30, but in others not, is not understood. Perhaps the specific type of pathogens that cause the immune reactions influence whether activated B cells turn on CD30 expression upon activation or not. The wide IGHV usage of the CD30^+^ B cells indicates that the antigenic trigger is quite diverse and not a unique type of pathogen. The immunohistochemical double stainings revealed a consistent lack of CD138 expression by the CD30^+^ cells in the lymph nodes with frequent CD30^+^ lymphocytes. This is in line with earlier studies on normal CD30^+^ B cells (2, 12) and shows that the cells are not plasma cells. IRF4 is frequently expressed by the CD30^+^ B cells, which was already reported (3, 13, 14). IRF4 is normally expressed by a subset of GC B cells and also plays a role in class switching (29). In the extrafollicular CD30^+^ B cells, expression of IRF4 may indicate a differentiation towards a plasmablast phenotype, as this transcription factor is expressed by plasmablasts and plasma cells.

The finding of small expanded clones among the CD30^+^ B cells in five of the eight lymph nodes studied is not unexpected, because CD30^+^ B cells in the GC are members of expanding GC B-cell clones, and also extra-follicular CD30^+^ B cells have proliferative features, as already mentioned (2, 14, 30). The intraclonal IGHV gene diversity present in several of the mutated clones (**Figure 3**) likely reflects that some of the clones may be members of expanding GC B-cell clones undergoing active somatic hypermutation during their clonal expansion. Other clones with intraclonal diversity may represent reactivated memory B cells that derive from a common GC B-cell clone that gave rise to multiple post GC B cells with substantial intraclonal diversity, as it is typical for human memory B-cell clones (31, 32).

In conclusion, we show here that CD30^+^ B cells in reactive lymph nodes with relatively high numbers of these cells are typically not monoclonal B-cell expansions as potential precursor lesions of HL. These cells are a diverse population, in all instances studied here being composed of activated and presumably proliferating naive B cells, GC B cells, and memory B cells in variable proportions. It remains to be clarified whether there are specific types of immune reactions that promote CD30 expression by (a fraction of) the activated B cells.

## Data availability statement

The IGHV gene sequences presented in the study are deposited in the Genbank repository (https://www.ncbi.nlm.nih.gov/gengank/), accession numbers OR076452-OR076692.

## Ethics statement

The studies involving human participants were reviewed and approved by Ethical committee of the Johann Wolfgang Goethe University of Frankfurt/Main, University Hospital, Frankfurt/Main, Germany. Written informed consent for participation was not required for this study in accordance with the national legislation and the institutional requirements.

## Author contributions

RK and M-LH conceived and designed the study. M-LH and SH selected tissue samples, evaluated immunohistochemical stainings and supervised the microdissection. RK supervised the PCR analyses. RK and BB evaluated IGHV gene sequences. RK wrote the paper. All authors read and approved the final manuscript.
