# Supplementary material for: Clonal composition and differentiation stage of human CD30+ B cells in reactive lymph nodes
Source: Front Immunol. 2023 Jul 25;14:1208610. doi: 10.3389/fimmu.2023.1208610 (PMC10407394; doi:10.3389/fimmu.2023.1208610)
Supplement: Supplementary file 1 [file Table_1.docx]

**Suppl. Table 1** Reactive lymph nodes studied.

| Case | Gender | Diagnosis | Distribution of CD30^+^ cells |
| --- | --- | --- | --- |
| 1 | f | chronic unspecific lymphadenitis | interfollicular |
| 2 | m | chronic unspecific lymphadenitis, including progressively transformed germinal centers | more interfollicular than in GC |
| 3 | f | chronic unspecific lymphadenitis | GC + interfollicular |
| 4 | m | lymphadenitis | GC + interfollicular |
| 5 | m | lymphadenitis | GC + interfollicular |
| 6 | f | chronic unspecific lymphadenitis | interfollicular |
| 7 | m | lymphadenitis | mainly in GC, few interfollicular |
| 8 | f | unspecific reactive reaction | interfollicular |

f, female; m, male

**Suppl. Table 2** IGHV gene analysis of single CD30^+^ B cells from case 1

| Cell | IGHV gene | IGHD gene | IGHJ gene | Productive? | Mutation frequency  (%) |
| --- | --- | --- | --- | --- | --- |
| 3 | 3-33  1-3 | 2-15  n.i. | 3  4 | yes  no (stop codon, oof) | 8.7  5.0 |
| 5 | 1-69 | n.i. | 6 | yes | 0 |
| 8 | 5-51  3-11 | 2-15  2-15 | 3  3 | yes  yes | 13.5  6.1 |
| 18 | 4-31  3-23 | 3-3  n.i. | 4  4 | no (stop codon, oof)  yes | 0  6.8 |
| 19 | 1-46 | 3-10 | 4 | yes | 4.3 |
| 24 | 4-30 | 2-8 | 5 | yes | 4.9 |
| 25 | 3-23 | 5-5 | 4 | yes (9 bp del) | 6.7 |
| 26 | 4-34 | 6-13 | 3 | yes | 0 |
| 27 | 3-11 | 1-7 | 4 | yes | 0 |
| 32 | 3-49 | n.i. | 4 | yes | 2.4 |
| 43 | 3-9 | 6-19 | 3 | yes | 0 |
| 44 | 2-5 | 6-19 | 4 | yes | 0 |
| 47 | 3-30 | n.i. | 6 | no (oof) | 0 |
| 52 | 3-21 | 1-7 | 4 | yes | 0 |
| 58 | 3-7 | 3-9 | 4 | yes | 0 |
| 60 | 4-30-2 | n.i. | 4 | yes | 0 |
| 75 | 3-15 | 3-22 | 4 | yes | 0 |
| 79 | 3-9 | n.i. | 6 | no (oof, deletions, duplication) | 11.9 |
| 83 | 3-21 | 3-9 | 5 | yes | 8.1 |
| 86 | 1-2 | 3-3 | 3 | yes | 5.6 |
| 87 | 3-48 | 3-3 | 4 | yes | 5.0 |
| 90 | 3-13 | 3-3 | 4 | no (oof) | 0 |
| 92 | 1-2 | 2-8 | 4 | yes | 0 |
| 104 | 4-39 | 3-10 | 3 | no (stop codon, oof) | 1 |
| 107 | 1-69 | 1-26 | 4 | yes | 0 |
| 111 | 3-13 | n.i. | 3 | yes | 0 |
| 119 | 3-30 | n.i. | 6 | yes | 9.8 |
| 120 | 3-13 | 6-19 | 6 | no (stop codon) | 0 |

n.i., not identifiable

oof, out-of-frame

**Suppl. Table 3** IGHV gene analysis of single CD30^+^ B cells from case 2

| Cell | IGHV gene | IGHD gene | IGHJ gene | Productive? | Mutation frequency (%) | Clone No. |
| --- | --- | --- | --- | --- | --- | --- |
| 3 | 3-21 | 4-17 | 4 | yes | 0 |  |
| 4 | 3-33 | 6-19 | 4 | yes | 0 |  |
| 7 | 3-30 | 5-12 | 4 | yes | 0 |  |
| 8 | 4-39 | 3-3 | 4 | yes | 2.5 |  |
| 10 | 3-23  1-3 | 3-22  2-15 | n.i.  4 | n.i.  if | 0.8  0.8 |  |
| 15 | 3-21 | 4-17 | 3 | yes | 0.5 |  |
| 18 | 4-39 | 2-15 | 5 | oof | 0 |  |
| 21 | 3-7  4-39 | n.i.  6-19 | 6  4 | yes  yes | 1.0  0 |  |
| 22 | 3-30 | 6-19 | 5 | yes | 7.0 | 3 |
| 24 | 1-18 | n.i. | 4 | yes | 0 |  |
| 32 | 3-11 | n.i. | 4 | yes | 2.9 | 2 |
| 33 | 1-2 | 3-3 | 4 | yes | 2.2 |  |
| 38 | 1-8 | n.i. | 4 | yes | 2.2 |  |
| 39 | 3-15 | 3-22 | 4 | yes | 3.2 |  |
| 41 | 1-46 | 2-21 | 4 | stop codon | 1.7 |  |
| 42 | 3-21 | 1-26 | 4 | yes | 4.4 |  |
| 43 | 4-34 | n.i. | 5 | yes | 0 |  |
| 46 | 1-46 | 2-8 | 3 | yes | 0 | 5 |
| 47 | 3-9 | 1-14 | 4 | yes | 1.0 | 1 |
| 59 | 1-18 | 3-10 | 5 | yes | 0 | 4 |
| 60 | 1-18 | 3-10 | 5 | yes | 0 | 4 |
| 61 | 3-33 | 1-26 | 4 | oof | 1.0 |  |
| 73 | 3-48 | 3-3 | 5 | oof | 3.4 |  |
| 77 | 3-11 | n.i. | 4 | yes | 3.1 | 2 |
| 82 | 3-9 | 1-14 | 4 | yes | 1.0 | 1 |
| 84 | 3-53 | 3-22 | 3 | yes | 3.9 |  |
| 87 | 1-24 | 2-2 | 3 | yes | 5.2 |  |
| 89 | 1-46 | 2-8 | 3 | yes | 0.5 | 5 |
| 101 | 3-30  1-69 | 6-19  1-26 | 5  4 | yes  yes | 6.0  6.6 | 3 |
| 106 | 5-51 | n.i. | 4 | yes | 0 |  |
| 107 | 4-39 | n.i. | 4 | yes | 1.0 |  |
| 110 | 3-23 | 3-10 | 4 | yes | 1.5 |  |
| 111 | 3-23 | 3-22 | 4 | yes | 0 |  |
| 114 | 4-39 | 3-3 | 5 | yes | 0 |  |
| 117 | 3-23 | n.i. | 4 | yes | 5.4 |  |
| 118 | 3-30 | 6-19 | 5 | yes | 8.0 | 3 |

n.i., not identifiable

oof, out-of-frame

**Suppl. Table 4** IGHV gene analysis of single CD30^+^ B cells from case 3

| Cell | IGHV gene | IGHD gene | IGHJ gene | Productive? | Mutation frequency (%) | Remarks |
| --- | --- | --- | --- | --- | --- | --- |
| 4 | 3-30 | n.i. | 6 | yes | 10.2 |  |
| 6 | 4-4 | 3-10 | 6 | yes | 1.0 |  |
| 8 | 4-30 | n.i. | 4 | yes | 8.4 |  |
| 16 | 4-34 | 3-10 | 4 | yes | 6.6 |  |
| 20 | 3-74 | 6-19 | 3 | yes | 5.4 |  |
| 21 | 3-30  4-59 | 5-24  2-2 | 6  6 | yes  yes | 6.0  0 |  |
| 25 | 1-46 | 3-22 | 4 | yes | 7.0 | 3 bp deletion CDR2 |
| 30 | 3-33 | 2-15 | 4 | yes | 7.3 |  |
| 32 | 3-38-3 | 6-13 | 6 | no (oof) | 0 |  |
| 34 | 1-2 | 1-7 | 3 | yes | 11.5 |  |
| 36 | 3-30 | n.i. | n.i. | yes | 5.6 |  |
| 38 | 3-30-3 | 2-15 | 3 | yes | 0.5 |  |
| 42 | 3-30 | 6-19 | 4 | yes | 8.2 |  |
| 48 | 4-61 | n.i. | 6 | yes | 10.9 | clone 1; 9 bp deletion CDR2 |
| 51 | 4-59 | 3-9 | 4 | yes | 7.0 |  |
| 70 | 5-51 | 2-2 | 3 | yes | 3.1 | amplified with VH1 primer |
| 83 | 3-66 | 3-3 | 4 | yes | 0 |  |
| 85 | 3-23 | 2-21 | 3 | yes | 5.4 |  |
| 86 | 1-2 | 1-7 | 3 | yes | 8.5 |  |
| 90 | 4-39 | 5-5 | 3 | yes | 1.5 |  |
| 91 | 3-23 | 2-8 | 3 | yes | 8.8 |  |
| 93 | 4-61 | n.i. | 6 | yes | 9.9 | clone 1; 9 bp deletion CDR2 |
| 99 | 1-69 | 3-22 | 3 | yes | 1.3 |  |
| 110 | 1-18  3-30  3-53 | 3-16  n.i.  3-10 | 4  3  1 | yes  yes  yes | 0  15.5  4.2 |  |
| 116 | 3-9 | 3-10 | 4 | yes | 10.0 |  |
| 117 | 3-30 | 2-15 | 5 | no (oof) | 5.4 | 6 bp deletion in FR3 |
| 118 | 3-30 | 4-4 | 5 | yes | 3.0 |  |
| 119 | 3-53 | n.i. | 6 | yes | 0 |  |

n.i., not identifiable

oof, out-of-frame

**Suppl. Table 5** IGHV gene analysis of single CD30^+^ B cells from case 4

| Cell | IGHV gene | IGHD gene | IGHJ gene | Productive? | Mutation frequency (%) | Remarks |
| --- | --- | --- | --- | --- | --- | --- |
| 9 | 3-7 | n.i. | 3 | yes | 10.0 |  |
| 13 | 3-30 | 2-2 | 4 | yes | 0 |  |
| 15 | 1-2 | 1-7 | 3 | yes | 14.0 | clone 1 |
| 16 | 1-2 | 1-7 | 3 | yes | 13.1 | clone 1 |
| 26 | 1-69 | 1-26 | 4 | yes | 0 |  |
| 27 | 3-23  4-34 | 4-17  2-21 | 4  3 | yes  yes | 13.5  0.5 |  |
| 36 | 1-2 | 1-7 | 3 | yes | 9.3 | clone 1 |
| 41 | 1-2 | 3-3 | 6 | no | 0 | stop codon, oof |
| 45 | 4-39 | 2-2 | 3 | yes | 0 |  |
| 47 | 3-11 | n.i. | 3 | yes | 9.7 |  |
| 49 | 3-74 | 6-6 | 6 | no | 0 |  |
| 60 | 1-2 | 1-7 | 3 | yes | 15.4 | clone 1 |
| 62 | 3-11 | 3-22 | 1 | no | 0 | stop codon, oof |
| 63 | 1-2 | 1-7 | 3 | no | 15.4 | clone 1, nonsense mutation |
| 75 | 3-7 | 3-22 | 4 | yes | 1.5 |  |
| 76 | 3-9 | 1-26 | 3 | yes | 3.4 |  |
| 77 | 3-33 | n.i. | 4 | yes | 0 |  |
| 80 | 4-34 | 3-22 | 4 | yes | 2.6 |  |
| 83 | 4-59 | 3-16 | 3 | yes | 0 |  |
| 86 | 3-30 | 2-2 | 4 | yes | 0 |  |
| 113 | 3-33 | 6-13 | 4 | yes | 7.8 |  |
| 136 | 1-69 | 6-19 | 3 | yes | 0 |  |
| 143 | 4-39 | 6-6 | 4 | no | 0 | stop codon, oof |
| 150 | 1-3 | n.i. | 6 | yes | 2.6 |  |

n.i., not identifiable

oof, out-of-frame

**Suppl. Table 6** IGHV gene analysis of single CD30^+^ B cells from case 5

| Cell | IGHV gene | IGHD gene | IGHJ gene | Productive? | Mutation frequency (%) | Remarks |
| --- | --- | --- | --- | --- | --- | --- |
| 7 | 3-30 | 2-21 | 5 | yes | 13.8 |  |
| 12 | 3-23 | 2-21 | 3 | yes | 5.5 | obtained with VH1 primer |
| 13 | 3-21 | n.i. | 6 | yes | 0 |  |
| 18 | 3-9 | n.i. | 3 | yes | 1.0 |  |
| 21 | 3-33 | 3-16 | 3 | yes | 0 |  |
| 31 | 1-69 | n.i. | 6 | yes | 3.1 |  |
| 57 | 4-34 | n.i. | 4 | yes | 0 |  |
| 58 | 3-23 | 3-10 | 4 | yes | 0 |  |
| 62 | 1-3 | 6-13 | 4 | no | 3.1 | stops in CDR3 |
| 88 | 3-33 | 3-9 - 3-10 | 4 | no | 0.5 | with IGHD3-9-IGHD3-10 |
| 92 | 3-33 | n.i. | 4 | yes | 2.5 |  |
| 97 | 3-7 | 3-9 | 4 | yes | 0 |  |
| 118 | 3-9 | 4-17 | 3 | yes | 10.3 |  |
| 119 | 3-48 | 2-2 | 4 | no (oof) | 0 |  |
| 120 | 4-34 | n.i. | n.i. | yes | 2.6 |  |
| 123 | 3-11 | 3-10 | 5 | yes | 0 |  |
| 132 | 1-69 | 3-10 | 3 | yes | 12.5 |  |
| 137 | 3-11 | 3-10 | 3 | yes | 7.0 |  |
| 157 | 1-2 | 3-9 | 4 | yes | 1.0 |  |
| 169 | 1-2 | n.i. | 1 | yes | 0 |  |
| 170 | 3-74 | n.i. | 4 | yes | 2.0 |  |
| 173 | 3-53 | n.i. | 1 | yes | 14.1 |  |
| 175 | 1-2 | n.i. | n.i. | yes | 0 |  |
| 179 | 3-7 | 1-26 | 3 | yes | 0 |  |
| 180 | 3-48 | 3-22 | 3 | yes | 0 |  |

n.i., not identifiable

oof, out-of-frame

**Suppl. Table 7** IGHV gene analysis of single CD30^+^ B cells from case 6

| Cell | IGHV gene | IGHD gene | IGHJ gene | Productive? | Mutation frequency (%) | Remarks |
| --- | --- | --- | --- | --- | --- | --- |
| 8 | 4-4 | 1-1 | 3 | yes | 7.9 | clone 1 |
| 21 | 1-69 | 4-11 | 4 | yes | 8.6 | 6 bp insertion in FR3 |
|  | 3-7 | 3-10 | 4 | no (oof) | 11.7 |  |
| 40 | 3-48 | 6-19 | 3 | yes | 8.5 |  |
| 45 | 3-53 | n.i. | 3 | yes | 8.5 |  |
| 61 | 3-15 | 1-26 | 5 | yes | 2.9 |  |
| 62 | 3-11 | n.i. | 4 | yes | 0 |  |
| 87 | 3-15 | n.i. | 4 | yes | 0 |  |
|  | 3-9 | n.i. | 6 | no (oof) | 0 |  |
| 95 | 3-33 | 1-20 | 3 | yes | 5.0 | clone 2 |
| 105 | 4-34 | 1-1 | 3 | yes | 10.5 |  |
| 142 | 4-31 | 3-22 | 4 | yes | 7.5 |  |
| 149 | 4-59 | 3-22 | 4 | yes | 3.6 |  |
| 151 | 4-4 | 1-1 | 3 | yes | 2.0 | clone 1 |
| 156 | 1-3 | 3-22 | 4 | no (oof & ns) | 0.5 |  |
| 168 | 3-30 | n.i. | 4 | yes | 0 |  |
| 179 | 1-8 | n.i. | 6 | yes | 0.9 |  |
| 180 | 1-24 | n.i. | 4 | yes | 0 |  |
| 185 | 3-33 | 3-22 | 4 | yes | 0 |  |
| 190 | 1-24 | 7-27 | 4 | yes | 6.5 |  |
| 198 | 3-33 | 1-20 | 3 | yes | 4.7 | clone 2 |
| 200 | 3-20 | 5-12 | 3 | yes | 11.3 |  |

n.i., not identifiable

ns, nonsense

oof, out-of-frame

**Suppl. Table 8** IGHV gene analysis of single CD30^+^ B cells from case 7

| Cell | IGHV gene | IGHD gene | IGHJ gene | Productive? | Mutation frequency (%) | Remarks |
| --- | --- | --- | --- | --- | --- | --- |
| 6 | 4-61 | 3-16 | 4 | yes | 2.8 |  |
| 10 | 1-69 | n.i. | 6 | yes | 3.1 |  |
| 15 | 1-58 | 5-12 | 4 | yes | 0 |  |
| 36 | 4-59 | 3-10 | 3 | yes | 0 |  |
| 37 | 3-33 | n.i. | 4 | yes | 2.0 |  |
| 39 | 4-39 | 5-24 | 3 | yes | 3.1 |  |
| 42 | 1-58 | 3-22 | 4 | yes | 0 |  |
| 44 | 1-69 | 5-24 | 4 | yes | 16.5 |  |
| 46 | 1-8 | 3-3 | 6 | yes | 4.3 |  |
| 47 | 1-69 | 1-26 | 4 | yes | 2.6 |  |
| 48 | 3-11 | 5-18 | 5 | yes | 7.0 |  |
| 55 | 4-39 | 3-22 | 3 | yes | 1.6 |  |
| 58 | 4-59 | n.i. | 3 | no (oof) | 1.0 |  |
| 103 | 1-69 | 3-22 | 3 | yes | 7.4 |  |
| 104 | 3-7 | 1-1 | 3 | yes | 0 |  |
| 117 | 3-66 | n.i. | 5 | yes | 3.0 |  |
| 130 | 1-18 | 2-2 & 3-22 | 4 | no (oof) | 14.4 | 3 and 26 bp deletions |
| 142 | 1-2 | 4-17 | 4 | yes | 1.0 |  |
| 146 | 1-2 | 6-19 | 4 | yes | 0 |  |
| 154 | 1-69 | 3-10 | 5 | yes | 13.3 |  |
| 162 | 5-51 | 3-22 | 3 | yes | 8.7 |  |
| 163 | 3-7 | 1-26 | 5 | yes | 0 |  |
| 167 | 3-48 | 2-15 | 4 | yes | 4.1 |  |
| 171 | 4-39 | 6-6 | 3 | yes | 0 |  |
| 178 | 4-59 | n.i. | 3 | yes | 7.2 |  |
| 181 | 1-69 | 5-12 | 4 | yes | 6.1 |  |
|  | 3-53 | 1-26 | 6 | yes | 0 |  |
| 182 | 1-69 | 6-19 | 4 | yes | 0 |  |
| 183 | 1-69 | n.i. | 6 | yes | 3.9 |  |
| 185 | 3-20 | n.i. | 3 | yes | 7.4 |  |
| 189 | 1-69 | 2-2 | 1 | yes | 0 |  |

n.i., not identifiable

oof, out-of-frame

**Suppl. Table 9** IGHV gene analysis of single CD30^+^ B cells from case 8

| Cell | IGHV gene | IGHD gene | IGHJ gene | Productive? | Mutation frequency (%) | Remarks |
| --- | --- | --- | --- | --- | --- | --- |
| 3 | 1-24 | 3-3 | 3 | yes | 7.4 |  |
| 4 | 3-23 | n.i. | 6 | yes | 8.5 | clone 1 |
| 7 | 3-33 | 4-17 | 4 | yes | 6.5 |  |
| 8 | 3-23 | 2-2 | 3 | yes | 8.5 |  |
| 10 | 3-30 | 4-17 | 6 | yes | 4.5 | clone 2 |
| 12 | 1-18 | 2-21 | 3 | yes | 0 |  |
| 13 | 3-30 | 1-26/5-12 | 1 | yes | 10.5 |  |
| 16 | 3-23 | 3-3 | 6 | yes | 0 |  |
| 17 | 1-24 | 2-2 | 6 | yes | 3.0 | clone 3 |
|  | 3-30 | 4-17 | 6 | yes | 5.0 | clone 2 |
| 18 | 1-8 | 5-12 | 6 | yes | 0.4 | clone 4 |
|  | 3-74 | 4-17 | 3 | yes | 8.0 |  |
| 24 | 5-10 | n.i. | 6 | yes | 1.5 |  |
| 28 | 4-39 | n.i. | 2 | yes | 3.6 |  |
| 29 | 3-23 | n.i. | 6 | yes | 8.3 | clone 1 |
| 34 | 3-23 | n.i. | 6 | yes | 8.8 | clone 1 |
| 38 | 3-7 | 2-2 | 3 | yes | 2.5 |  |
| 39 | 1-18 | n.i. | 1 | yes | 5.2 |  |
|  | 3-30-3 | 1-20 | 3 | yes | 5.9 |  |
| 47 | 3-23 | 2-2 | 3 | yes | 8.5 |  |
| 48 | 3-48 | 6-13 | 4 | yes | 0 |  |
| 51 | 3-7 | 3-3 | 6 | yes | 6.1 |  |
| 54 | 3-7 | n.i. | 6 | yes | 0 |  |
| 61 | 3-30 | 1-1/1-7 | 4 | yes | 19.0 | clone 5 |
| 63 | 4-34 | 2-8 | 6 | yes | 6.3 |  |
| 64 | 3-30 | 1-1/1-7 | 4 | yes | 18.2 | clone 5 |
| 65 | 3-30 | 2-8 | 3 | yes | 0 |  |
| 66 | 3-66 | 2-2 | 6 | yes | 0.5 |  |
| 67 | 3-13 | 2-21 | 4 | no | 0 | stop in CDR3 |
| 68 | 3-48 | n.i. | 6 | yes | 12.2 |  |
| 75 | 3-9 | 5-18 | 4 | yes | 2.0 |  |
| 94 | 3-15 | 3-10 | 4 | no | 0 | oof |
| 97 | 3-30 | n.i. | 3 | yes | 8.3 |  |
| 100 | 3-21 | n.i. | 1 | yes | 0 |  |
| 110 | 1-8 | 5-12 | 6 | yes | 0.5 | clone 4 |
| 114 | 1-24 | 2-2 | 6 | yes | 3.0 | clone 3 |
|  | 3-7 | n.i. | 6 | yes | 1.0 |  |

n.i., not identifiable

oof, out-of-frame
